# Supplementary material for: Computationally-driven engineering of sublattice ordering in a hexagonal AlHfScTiZr high entropy alloy
Source: Sci Rep. 2017 May 19;7:2209. doi: 10.1038/s41598-017-02385-w (PMC5438366; doi:10.1038/s41598-017-02385-w)
Supplement: Supplementary file 1 — Mechanical tensile tests [file 41598_2017_2385_MOESM1_ESM.doc]

**Supplementary material**

Computationally-driven engineering of sublattice ordering in a hexagonal AlHfScTiZr high entropy alloy

Lukasz Rogal^1a^, Piotr Bobrowski^1^, Fritz Körmann^2^, Sergiy Divinski^4^, Frank Stein^3^, Blazej Grabowski^3b^

*^1^Institute of Metallurgy and Materials Science of the Polish Academy of Sciences, 30-059 Krakow, Poland*
*^2^Materials Science and Engineering, Delft University of Technology, 2628 CD Delft, Netherlands*
*^3^Max-Planck-Institut für Eisenforschung GmbH D-40237 Düsseldorf, Germany*

*^4^Institute of Materials Physics, University of Münster, Wilhelm-Klemm-Str. 10, 48149 Münster, Germany*

*Corresponding authors: ^a)^L. Rogal, l.rogal@imim.pl, tel.+48 122952801, fax +48 122952804*

*^b)^ Blazej Grabowski,* [*b.grabowski@mpie.de*](mailto:b.grabowski@mpie.de)*, tel:+49 211 6792 512, fax:+492116792512*

**Micro-mechanical tests for Al_15_Hf_25_Sc_10_Ti_25_Zr_25_ at.%**

Tensile tests were performed on a custom-built device. Dogbone-shaped samples with a gauge length of 3 mm and 1.0x0.3 mm^2^ were cut by spark erosion from as-cast samples and from a sample after thermal treatment at 1000°C for 5h.

The results of the tensile tests are shown in Fig. S1. In the as-cast state (red line) the yield strength approaches a value of about 1400 MPa and the elongation to failure (excluding the elastic part) is about 0.5%, Fig. S1. As for the annealed state, two samples were investigated (green and blue curves) to improve statistics. Both samples revealed brittle fracture at a stress of about 1000 MPa.

| 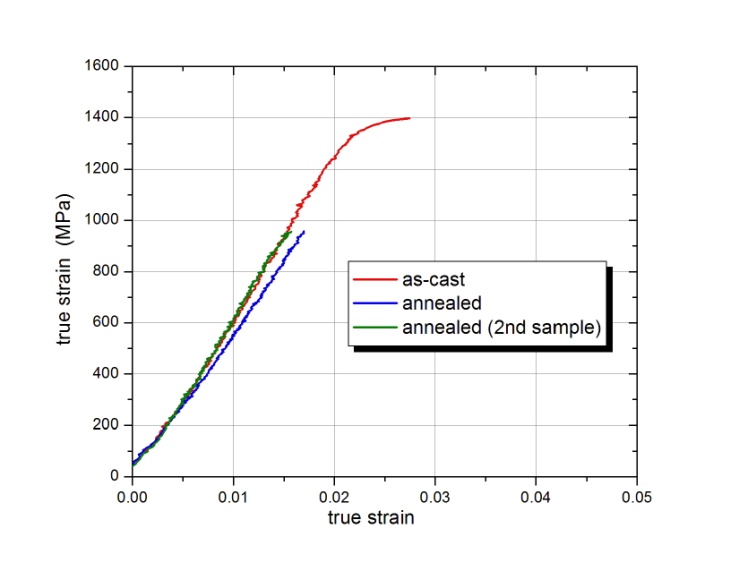 | **Figure S1**. True stress-true strain tensile curves for the as-cast (red line) and annealed (green and blue lines) Al_15_Hf_25_Sc_10_Ti_25_Zr_25_ at.% HEA. |
| --- | --- |

Although our custom-built device does not provide correct values of the elastic modulus, the yield strength and elongation to failure are reliable [1].

The fracture surfaces were examined by a FEI NanoSEM 230 field-emission-gun scanning electron microscope (FEG-SEM). The results are presented in Fig. S2. The as-cast sample reveals a pronounced ductile fracture with a combination of transgranular and intergranular features and a developed vain-like structure, Fig. S2a and b. In contrast, the annealed samples reveal brittle fracture with predominantly transgranular character, Fig. 2c, d. Nevertheless Fig. 2c and d document a pronounced dislocation activity during fracture of the ordered Al_15_Hf_25_Sc_10_Ti_25_Zr_25_ at.% HEA.

| a) 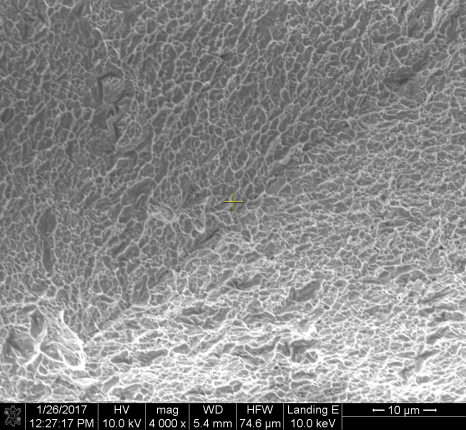 | b) 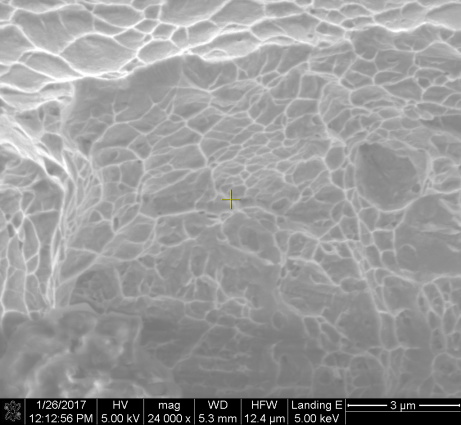 |
| --- | --- |
| c) 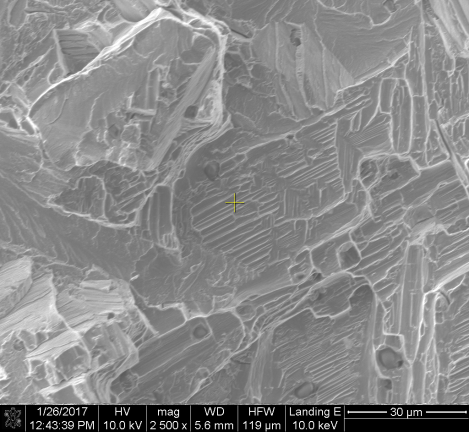 | d)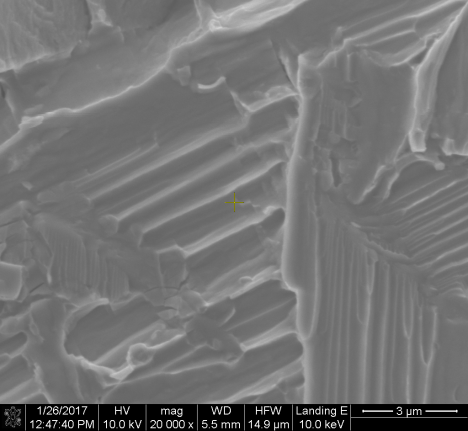 |

**Figure S2**: SEM images of the fracture surfaces observed on the as-cast (a, b) and annealed (c, d) Al_15_Hf_25_Sc_10_Ti_25_Zr_25_ at.% HEAs.

[1] J. Leuthold, *Mechanisms of time dependent plasticity in ultra-fine grained copper after severe plastic deformation*, PhD Thesis, Münster University (Germany) 2016.
